# Paternity in male kidney transplant recipients: a French national survey, the PATeRNAL study

**DOI:** 10.1186/s12882-020-02115-x

**Published:** 2020-11-16

**Authors:** Annabel Boyer, Thierry Lobbedez, Mohamed Ouethrani, Angélique Thuillier Lecouf, Nicolas Bouvier, Valérie Châtelet, Bruno Hurault de Ligny, On behalf of the Spiesser transplant group, Pierre-François Weestel, Jean-François Augusto, Yann Le Meur, Cyril Garrouste, Jean-Philippe Rérolle, Eric Thervet, Dany Anglicheau, Antoine Thierry, Charlotte Colosio, Joseph Rivalan, Isabelle Etienne, Sophie Caillard, Mathias Büchler

**Affiliations:** 1 Centre Universitaire des Maladies Rénales, CHU de Caen, Avenue de la côte de Nacre, 14033 Caen, Cedex 9 France; 2 U1086 INSERME – ANTICIPE, Centre Régional de Lutte contre le Cancer, François Baclesse, 14076 Caen, Cedex 5 France; 3 Unicaen, UFR de Médecine, Normandie Université, 2 rue des Rochambelles, 14032 Caen, Cedex France

**Keywords:** Paternity, Kidney transplant, Pregnancy, Congenital malformation, Immunosuppression

## Abstract

**Background:**

There is concern about the impact of immunosuppressive agents taken by male kidney transplant (KT) recipients on the risk of foetal malformations. The aim of our survey was to estimate the paternity rate and the outcomes of pregnancies fathered by kidney transplanted males.

**Methods:**

This survey analysed 1332 male KT recipients older than 18 years, followed in 13 centres in France. A self-reported questionnaire was used to collect data on the patients, treatments at the time of conception and the pregnancy outcomes.

**Results:**

The study included data on 349 children from 404 pregnancies fathered by 232 male KT recipients. The paternity rate was 17% (95% CI [15–20]). There were 37 (9%, 95% CI [7–12]) spontaneous abortions, 12 (3%, 95% CI [2–5]) therapeutic abortions, 2 (0.5%, 95% CI [0.1–1]) still births, and 13 (4%, 95% CI [2–6]) malformations reported. Compared to the general population, there was no difference in the proportion of congenital malformations nor unwanted outcomes whether the father was exposed or not to immunosuppressive agents.

**Conclusions:**

This survey does not provide any warning signal that pregnancies fathered by male patients exposed to immunosuppressive agents, notably the debated MMF/MPA, have more complications than pregnancies in the general population.

**Supplementary Information:**

**Supplementary information** accompanies this paper at 10.1186/s12882-020-02115-x.

## Background

The first child of a kidney transplant recipient was born in 1958. Pregnancy is currently considered one of the benefits accorded to women by kidney transplantation [1]. Despite early concerns about the teratogenicity of immunosuppressive medication, thousands of solid organ transplant recipients worldwide have had successful pregnancies after transplantation [2–9]. In 1991, the National Transplantation Pregnancy Registry (NTPR) was created to collect information on the outcomes of pregnancies among transplant recipients in North America [10].

Pregnancy in transplanted patients remains a challenge because of the increased risk of adverse maternal complications and adverse foetal outcomes [2–4, 7–9, 11]. In addition, immunosuppressive medications, such as sirolimus and mycophenolate mofetil/mycophenolic acid (MMF/MPA), have been associated with an increased incidence of foetal malformations [5, 6, 12–16]. A specific pattern of malformation has been described with MMF/MPA exposure during pregnancy, including microtia, cleft lip and palate, and congenital heart defects [4, 6, 13–17]. These data led the Food and Drug Administration to change the pregnancy category of MMF/MPA from C to D (positive evidence of human foetal risk, potential benefits may warrant its use despite the potential risk) in October 2007 [18]. There are guidelines for female kidney transplant (KT) recipients regarding the timing of pregnancy and the management of immunosuppressive therapy. MMF/MPA and sirolimus should be discontinued and replaced by another drug at least six weeks prior to conception [5, 7, 12, 19–21].

Information on fertility and the outcomes of pregnancies fathered by male KT recipients is sparse [4, 5, 22–24]. Some clinicians have expressed concern about the effect of immunosuppressive agents taken by the father at the time of conception on the risk of foetal malformation. Data from the NTPR are reassuring since the outcomes of pregnancies fathered by male KT recipients were similar to those from the general population [5, 22]. No pattern of malformation was observed in the offspring of 152 male recipients who were on MMF/MPA at the time of conception [4, 22]. In two population-based studies conducted in Norway, the risk of malformation was not increased when the father was on MMF/MPA at the time of conception [23, 24]. There is a lack of data regarding the fertility rate in men under immunosuppressive therapy after a kidney transplantation. A higher incidence of infertility in transplanted patients on sirolimus has been observed [4, 25–29].

Based on in vitro studies [30], the European Medicines Agency (EMA), and hence the French Agence Nationale de la Santé et des Médicaments (ANSM), have recommended since October 2015 that sexually active men treated with MMF/MPA must use effective contraception until at least 90 days after the cessation of MMF/MPA [31–33]. It was initially recommended that their female partners also use effective contraception during the same period. Since 2017, it is no longer advocated that both partners use contraception [34]. This recommendation is a matter of concern for the transplant teams and for the transplanted patients as planned fatherhood would require changing MMF/MPA for another immunosuppressive agent, which could increase the risk of rejection. It also raises the question of pregnancy termination in cases of paternal exposure to MMF/MPA. In the absence of scientific evidence, some authors urged the EMA to reconsider their position, or provide data to support it [35–37]. The current information in the literature is insufficient to allow institutions to make evidence-based decisions and thus additional studies are needed.

In the absence of reliable data, we aimed to conduct a nationwide survey of male transplanted patients followed in 13 centres in France. This survey was carried out to determine the paternity rate of male transplanted patients and to estimate the outcomes of pregnancies fathered by male KT recipients under immunosuppressive therapy at the time of conception.

## Methods

### Study population

This national survey was conducted by the Spiesser transplant group in France, which was created in 1997 and includes 13 of the 31 adult transplant centres in order to develop research protocols and share experiences in the field of renal transplantation. All male adults older than 18 years who lived in France and had received at least one kidney transplant in one of the participating centres between the January 1, 2005, and December 31, 2014, were included. Patients older than 60 years at the time of first transplantation, under guardianship, multi-organ transplanted or those who refused to participate were excluded from the survey.

### Objectives

The survey was carried out to seek for a signal of increased adverse pregnancy outcomes across male KT participants. Thus, the main objective was to determine the paternity rate in our population. The secondary objectives were to describe the outcome of pregnancies fathered by KT recipients under immunosuppressive therapy at the time of conception, and to investigate a possible future desire to father a child.

### Method

The survey was distributed by post between January 2018 and February 2019, with a pre-stamped return envelope, to male transplanted patients included in the study. A second dispatch was sent out to increase the number of responders. Two months after the first and second dispatches, a relaunch was sent to patients who had not responded, to maximise the number of participants.

A self-reported questionnaire, developed for this survey and adapted with authorization from the NTPR questionnaire, was used [38]. The questionnaire was divided into four sections and contained 32 items (Supplementary data, Figure S1). The collected data included global information on the patient: age, reference centre and year of kidney transplantation, number of children, and number of children after transplantation. If the patient did not have children, we collected information on the reasons why: anxiety, infertility, no desire. If the patient had children after transplantation, the collected data included: the year of pregnancy, the immunosuppressive agents taken at the time of conception and the pregnancy outcomes (miscarriage, stillbirth, livebirth, birth term, birth weight, congenital malformation, cognitive impairment). The last section of our questionnaire collected data on a possible future desire to father a child.

### Statistical analysis

Continuous variables are described by their median and interquartile range (IQR). Categorical variables are described by frequencies, percentage and their 95% confidence interval (95% CI). Missing data on our primary objective were less than 5% and were considered missing at random. Missing data on the other variables were as follow: less than 5% on patients’ characteristics, treatment at the time of conception and pregnancy outcomes (living birth, spontaneous abortion, therapeutic abortion, stillbirth, malformation); and 8, 11 and 12% on the birth weight, gestational age and year of pregnancy respectively. Analyses were performed with R 3.4.3 (R Foundation for Statistical Computing, Vienna, Austria).

The study was approved by the ethics committee of CHU de Caen and was conducted according to the declaration of Helsinki.

## Results

### Participants characteristics

**Fig. 1:**
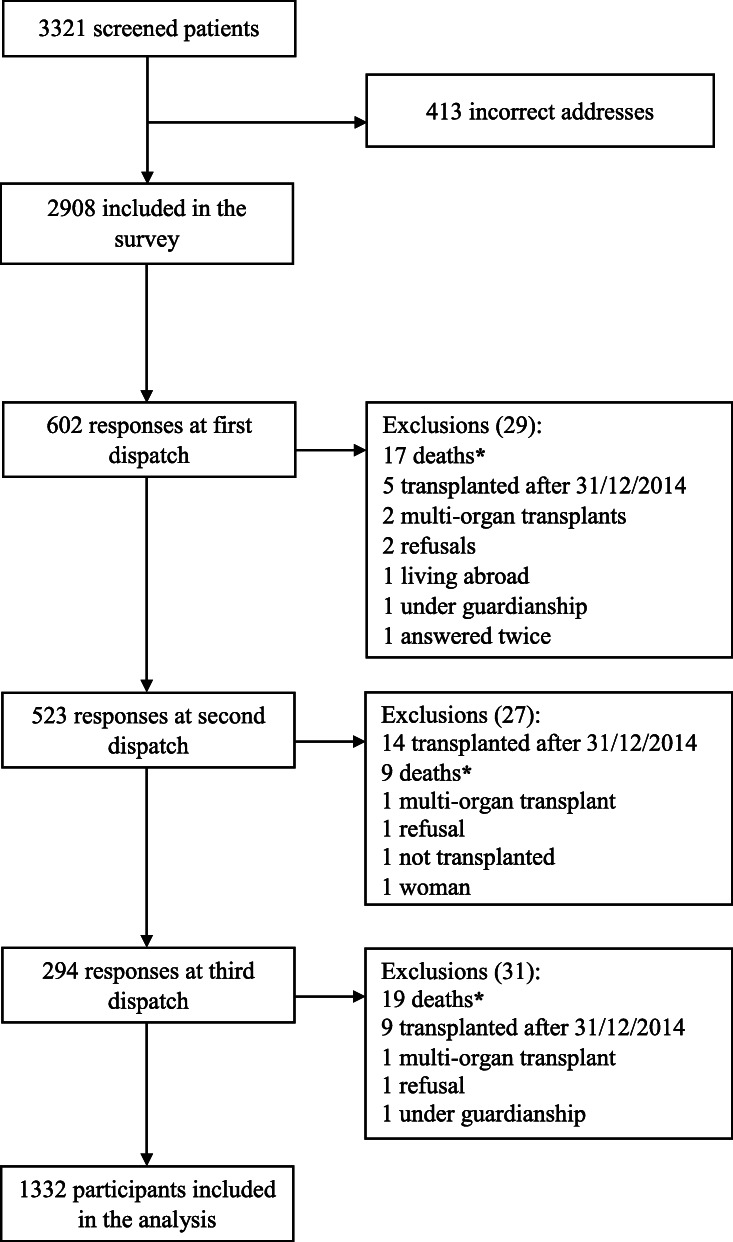
Flow chart. * Surveys were sent back by family members, with a notice of the patients’ recent death

Of the 3321 eligible male KT recipients, there were 413 (12%) missing addresses. Among the 2908 subjects who received the questionnaire, 1332 completed the survey (46% respond rate) (Fig. 1). Supplementary data Table S1 details the number of participants per centre. Participants with and without children were distributed equally across the 13 centres. Our population median age was of 55 years old [IQR 46–62]. The participants’ characteristics are described in Supplementary data Table S2.

### Paternity rate

With 232 participants having at least one child after transplantation, the paternity rate in our population was 17% (95% CI [15–20]), with on average 2 children after renal transplantation. Of these, 62 (27%, 95% CI [21–33]) male KT recipients had experienced fertility issues that were solved with medically assisted reproduction. A total of 1100 (83%, 95% CI [80–85]) participants did not have a child after renal transplantation: 330 (25%, 95% CI [22–27]) never had a child at all, and 770 (58%, 95% CI [55–60]) had children before transplantation only.

### Participants without children after transplantation

Of the 330 participants who never had a child, 105 (32%, 95% CI [27–37]) declared that they did not want to conceive after transplantation, 6 (2%, 95% CI [0.7–4]) male KT recipients were concerned about the risk of transmission of polycystic kidney disease (PKD), whereas 55 (17%, 95% CI [13–21]) suffered from infertility (Table 1). Childless participants seemed to be younger than the ones who had fathered children before KT only, with a median age of 50 years [IQR 39–59] and 59 years [IQR 53–64] respectively (Table 1).

**Table 1:** Description of participants without children

Covariate	Childless patients (***n*** = 330)
Age, median (IQR), years	50 (39–59)
Year of first transplantation, median (IQR)	2009 (2005–2012)
Reason, n (%, [95% CI])	
No desire to father a child	105 (32%, [27–37])
Infertility	55 (17%, [13–21])
Erectile dysfunction	5 (1%, [0.5–4])
Spermatogenesis disorders	33 (10%, [7–14])
Other	17 (5%, [3–8])
Anxiety	37 (11%, [8–15])
Other	
Age	7 (2%, [1–4])
Celibacy	75 (23%, [18–28])
Genetic PKD	6 (2%, [0.7–4])
Genetic disease other than PKD	11 (3%, [2–6])
Intellectual disability	4 (1%, [0.3–3])
Partner’s infertility	3 (1%, [0.2–3])
Missing	27 (8%, [5–12])
Ongoing pregnancy, n (%, [95% CI])	4 (1%, [0.3–3])

*IQR* interquartile range, *PKD* polycystic kidney disease

### Outcome of pregnancies fathered by male kidney transplant recipients under immunosuppressive therapy at the time of conception

There were 349 children from 404 pregnancies fathered by 232 male KT recipients, including twin pregnancies. Unwanted outcomes occurred in the 404 pregnancies as follows: 37 (9%, 95% CI [7–12]) spontaneous abortions, 12 (3%, 95% CI [2–5]) therapeutic abortions, and 2 (0.5%, 95% CI [0.1–1]) still births. There were 10 (2%, 95% CI [1–5]) ongoing pregnancies at the time of the survey. Of the 349 living births, the median gestational age was 41 weeks (IQR [39–41]), with only 30 (9%, 95% CI [6–12]) pre-term deliveries. The median birth weight was 3.4 Kg (IQR [3–3.7]). There were 13 (4%, 95% CI [2–6]) congenital malformations reported (Table 2).

**Table 2:** Description and outcomes of pregnancies fathered by male kidney transplant patients

Covariate	All pregnancies ***n*** = 404	First pregnancy ***n*** = 245	Second pregnancy ***n*** = 115	Third pregnancy ***n*** = 35	Fourth pregnancy ***n*** = 9
Age, median (IQR), years	41 (36–48)	41 (36–48)	42 (37–48)	43 (37.5–49)	48 (44–52)
Age at conception, median (IQR), years	34 (30–37)	33 (21–36)	35 (31–39)	37 (34–40)	41 (40–44)
Year of first transplantation, median (IQR)	2006 (1999–2010)	2006 (1999–2010)	2006 (1996–2008)	2005 (1994–2007)	1994 (1986–2002)
Transplant to conception time in years, median (IQR)	5 (2–8)	4 (1.5–7)	7 (4–10)	8 (6–11)	16 (13–19)
Immunosuppressive co-medication at the time of conception^a^					
Corticosteroids	298 (74%, [69–78])	176 (72%, [66–77])	84 (73%, [64–81])	29 (83%, [66–93])	9 (100%, [66–100])
Azathioprine	59 (15%, [11–18])	44 (18%, [13–23])	13 (11%, [6–19])	1 (3%, [0.7–15])	1 (11%, [0.3–50])
MMF/MPA	313 (77%, [73–81])	190 (78%, [72–83])	92 (80%, [72–87])	26 (74%, [57–88])	5 (56%, [21–86])
Tacrolimus	255 (63%, [58–68])	156 (64%, [57–70])	70 (61%, [51–70])	22 (63%, [45–79])	7 (78%, [40–97])
Cyclosporine	152 (38%, [33–43])	94 (38%, [32–45])	43 (37%, [29–47])	13 (37%, [21–55])	2 (22%, [3–60])
Everolimus	5 (1%, [0.5–3])	3 (1%, [0.3–4])	2 (2%, [0.2–6])	0 (0%, [0–10])	0 (0%, [0–33])
Sirolimus	7 (2%, [1–4])	5 (2%, [0.7–5])	2 (2%, [0.2–6])	0 (0%, [0–10])	0 (0%, [0–33])
Belatacept	6 (1%, [0.5–3])	4 (2%, [0.4–4])	1 (1%, [0.1–5])	1 (3%, [0.7–15])	0 (0%, [0–33])
Other^b^	40 (10%, [7–13])	18 (7%, [4–11])	16 (14%, [8–22])	5 (14%, [5–30])	1 (11%, [0.3–50])
Rejection episode before or during pregnancy	80 (20%, [16–24])	40 (16%, [12–22])	29 (25%, [18–34])	8 (23%, [10–40])	3 (33%, [7–70])
Pregnancy outcomes					
Live births, including 6 twin pregnancies	349 (86%, [83–90])	222 (91%, [86–94])	94 (82%, [73–88])	26 (74%, [57–88])	7 (78%, [40–97])
Twin pregnancy	7 (2%, [0.5–3])	5 (2%, [0.7–5])	1 (1%, [0.1–5])^c^	1 (3%, [0.7–15])	0 (0%, [0–33])
Spontaneous abortion	37 (9%, [7–12])	18 (7%, [4–11])	14 (12%, [7–20])	4 (11%, [3–27])	1 (11%, [0.3–50])
Therapeutic abortion	12 (3%, [2–5])	6 (2%, [0.9–5])	2 (2%, [0.2–6])	3 (9%, [2–23])	1 (11%, [0.3–50])
Still births	2 (0.5%, [0.1–1])	0 (0%, [0–0.1])	0 (0%, [0–3])	2 (6%, [1–19])	0 (0%, [0–33])
Ongoing pregnancy	10 (2%, [1–5])	4 (2%, [0.4–4])	5 (4%, [1–10])	1 (3%, [0.7–15])	0 (0%, [0–33])
Live births, including twin pregnancies	349	222	94	26	7
Gestational age in weeks, median (IQR)	41 (39–41)	41 (38–41)	41 (39–41)	41 (39–41)	41 (39–41)
Pre-term delivery (< 37 weeks)	30 (9%, [6–12])	23 (10%, [7–15])	5 (5%, [2–12])	2 (8%, [1–25])	0 (0%, [0–41])
Birthweight (Kg), median (IQR)	3.4 (3–3.7)	3.3 (3–3.7)	3.4 (3–3.8)	3.5 (3–3.6)	3.5 (3.2–3.8)
Low birthweight (< 2.5 Kg)	20 (6%, [4–9])	16 (7%, [4–11])	3 (3%, [1–9])	1 (4%, [0.1–20])	0 (0%, [0–41])
Sex (male)	193 (55%, [50–61])	127 (57%, [50–64])	47 (50%, [40–60])	16 (62%, [41–80])	3 (43%, [10–82])
Intellectual disability	3 (1%, [0.2–3])	3 (1%, [0.3–4])	0 (0%, [0–4])	0 (%, [0–13])	0 (0%, [0–41])
Congenital malformation	13 (4%, [2–6])	10 (5%, [2–8])	3 (3%, [1–9])	0 (%, [0–13])	0 (0%, [0–41])

Values are presented as numbers and percentages [95% CI] unless otherwise specified

*IQR* interquartile range, *MMF/MPA* mycophenolate mofetil/mycophenolic acid, *Kg* Kilograms

^a^ Immunosuppressive agents of the father at the time of conception, more than one co-medication possible

^b^ Other: Immunosuppression including one of the following treatment before or at the time of conception: Cyclophosphamide, Intravenous immunoglobulin, Plasma exchange, Rituximab

^c^ The twin pregnancy was still ongoing at the time of the study

The outcomes of pregnancies fathered by male KT recipients according to the father’s immunosuppressive medication at the time of conception are described in Tables 3 and 4. The immunosuppressive agents and the malformations or therapeutic abortions are displayed in Table 5. Since before the pregnancies the male patients were exposed to co-medication, outcomes can be declared in several columns. Of the 5 pregnancies within the partnerships of the participants treated by everolimus, there was one therapeutic abortion due to multiple malformations. The proportion of therapeutic abortions and malformations according to the immunosuppressive agents taken by the father were: 6 of 59 (10%, 95% CI [4–21]) pregnancies for azathioprine (AZA), 18 of 255 (7%, 95% CI [4–11]) pregnancies for tacrolimus, 19 of 298 (6%, 95% CI [4–10]) pregnancies for corticosteroid (CTC), 17 of 313 (5%, 95% CI [3–9]) pregnancies for MMF/MPA, and 6 of 152 (4%, 95% CI [1–8]) pregnancies for cyclosporine. The most common treatment associations were: CTC – MMF/MPA – tacrolimus (159 pregnancies conceived by 103 patients) and CTC – MMF/MPA – cyclosporine (72 pregnancies conceived by 45 patients), with a malformation rate of 3.7% (95% CI [1.5–8]) and 1.4% (95% CI [0.7–8.5]) respectively.

**Table 3:** Description and outcomes of pregnancies fathered after kidney transplantation according to the fathers’ immunosuppressive medication at the time of conception

Covariate	MMF/MPA ***n*** = 313	CTC ***n*** = 298	Tacrolimus ***n*** = 255	Cyclosporine ***n*** = 152
Number of recipients	195	177	166	94
Age, median (IQR), years	40 (36–45)	43 (37–50)	40 (35–45)	46 (38–52)
Age at conception, median (IQR), years	34 (30–38)	34 (31–38)	35 (31–38)	33 (29–37)
Transplant to conception time^a^, median (IQR)	5 (2–9)	5 (3–10)	5 (7–10)	4 (2–7.5)
Pregnancy outcomes				
Live births, including 6 twin pregnancies	268 (86%, [81–89])	257 (86%, [82–90])	219 (86%, [81–90])	136 (89%, [83–94])
Twin pregnancy	6 (2%, [0.7–4])^b^	6 (2%, [0.7–4])^b^	5 (2%, [0.6–5])	2 (1%, [0.2–5])^b^
Spontaneous abortion	32 (10%, [7–14])	29 (10%, [7–14])	23 (9%, [6–13])	12 (8%, [4–13])
Therapeutic abortion	8 (3%, [1–5])	10 (3%, [2–6])	8 (3%, [1–6])	2 (1%, [0.2–5])
Still births	2 (1%, [0.1–2])^c^	2 (0.7%, [0.1–2])^c^	2 (0.8%, [0–3])^c^	0 (0%, [0–2])
Ongoing pregnancy	8 (3%, [1–5])	5 (2%, [0.5–4])	8 (3%, [1–6])	3 (2%, [0.4–6])
Live births, including twins	268	257	219	136
Gestational age in weeks, median (IQR)	41 (39–41)	41 (39–41)	41 (39–41)	41 (39–41)
Pre-term delivery (< 37 weeks)	24 (9%, [6–13])	22 (9%, [5–13])	16 (7%, [4–12])	16 (12%, [7–18])
Birthweight (Kg), median (IQR)	3.3 (3–3.7)	3.4 (3–3.7)	3.4 (3–3.7)	3.3 (2.9–3.7)
Low birthweight (< 2.5 Kg)	18 (7%, [4–10])	10 (4%, [2–7])	12 (5%, [3–9])	9 (7%, [3–12])
Sex (male)	142 (53%, [47–59])	143 (56%, [49–62])	119 (54%, [47–61])	76 (56%, [47–64])
Intellectual disability	1 (0.4%, [0–2])	2 (0.8%, [0–3])	1 (0.5%, [0–3])	1 (0.7%, [0–4])
Congenital malformation	9 (3%, [1–6])	9 (4%, [2–7])	10 (5%, [2–8])	4 (3%, [1–7])

Values are presented as numbers and percentages [95% CI] unless otherwise specified

As before the pregnancies the male patients were exposed to co-medication, one outcome can be declared in several columns

*MMF* Mycophelonate mofetil, *CTC* corticosteroid, *IQR* interquartile range

^a^ Transplant to conception time, in years

^b^ One of the twin pregnancy was still ongoing at the time of the study

^c^ In both stillbirths, from a twin pregnancy, a pulmonary malformation was discovered

**Table 4:** Description and outcomes of pregnancies fathered after kidney transplantation according to the fathers’ immunosuppressive medication at the time of conception

Covariate	Azathioprine ***n*** = 59	Sirolimus ***n*** = 7^b^	Belatacept ***n*** = 6^b^	Everolimus ***n*** = 5^b^
Number of recipients	46	7	4	5
Age, median (IQR), years	51 (44–56)	40 (38–48)	39 (35–42)	55 (37–62)
Age at conception, median (IQR), years	34 (29–36)	34 (29–36)	38 (35–39)	37 (34–40)
Transplant to conception time^a^, median (IQR)	5 (2–9.5)	4 (1.5–12)	6 (5–8)	7 (4–10.8)
Pregnancy outcomes				
Live births, including 6 twin pregnancies	55 (93%, [84–98])	5	4	5
Twin pregnancy	1 (2%, [0–9])	0	0	1
Spontaneous abortion	2 (3%, [0.5–11])	2	2	0
Therapeutic abortion	2 (3%, [0.5–11])	0	0	1
Still births	0 (0%, [0–6])	0	0	0
Ongoing pregnancy	1 (2%, [0–9])	0	0	0
Live births, including twins	55	5	4	5
Gestational age in weeks, median (IQR)	41 (39–41)	39 (38–40)	38 (38–39)	39 (36–41)
Pre-term delivery (< 37 weeks)	5 (9%, [3–20])	0	0	2
Birthweight (Kg), median (IQR)	3.5 (3.1–3.6)	3 (2.4–3.3)	3.1 (2.9–3.2)	3.2 (2.9–3.6)
Low birthweight (< 2.5 Kg)	1 (2%, [0.1–10])	1	0	0
Sex (male)	33 (60%, [46–73])	3	2	3
Intellectual disability	2 (4%, [0.5–13])	0	0	0
Congenital malformation	4 (7%, [2–17])	0	0	0

Values are presented as numbers and percentages [95% CI] unless otherwise specified

As before the pregnancies the male patients were exposed to co-medication, one outcome can be declared in several columns

*MMF* Mycophelonate mofetil, *IQR* interquartile range

^a^ Transplant to conception time, in years

^b^ Due to the small number of events, percentages are not presented

**Table 5:** List of therapeutic abortions and congenital malformations identified among 25 offspring of male kidney transplant recipients exposed to immunosuppressive medication

**Therapeutic abortion,** ***n*** **= 12**				
Year of pregnancy	Year of transplant	Paternal age^a^	Medical reason for abortion	Male KT recipients’ medication regimen at the time of conception
1986	1981	NA	Genetic disorder	AZA, prednisone
NA	1994	NA	Trisomy 21	MMF/MPA, tacrolimus, prednisone
2005	1979	31	Spina bifida and Arnold Chiari	AZA, prednisone
2012	2006	48	Ectopic pregnancy	MMF/MPA, cyclosporine
2013	2007	34	Trisomy 21	MMF/MPA, cyclosporine
2014	2010	36	missing	MMF/MPA, tacrolimus, prednisone
2015	2006	34	missing	MMF/MPA, tacrolimus, prednisone
2016	2009	40	missing	MMF/MPA, tacrolimus, prednisone
2016	2008	35	missing	Tacrolimus, prednisone
2016	2011	35	Multiple malformations	Tacrolimus, everolimus, prednisone
2018	2014	33	missing	MMF/MPA, tacrolimus, prednisone
2018	2013	43	missing	MMF/MPA, tacrolimus, prednisone
**Congenital malformation,** ***n*** **= 13**				
Year of birth	Year of transplant	Paternal age^a^	Congenital malformation	Male KT recipients’ medication regimen at the time of conception
1998	1996	33	Cleft lip and palate	AZA, cyclosporine
2000	NA	NA	Deafness	AZA, cyclosporine, prednisone
2001	1998	32	Congenital phimosis	MMF/MPA, cyclosporine, tacrolimus, prednisone
2001	2000	NA	Cleft lip and palate	AZA, tacrolimus, prednisone
2002	1989	41	Complex cardio-facial syndrome	AZA, tacrolimus, prednisone
2005	2004	34	Left thumb’s agenesis	MMF/MPA, tacrolimus, prednisone
2006	1992	25	Pyloric stenosis	MMF/MPA, tacrolimus, prednisone
2009	2007	21	Renal hypoplasia	MMF/MPA, tacrolimus
2012	2007	30	Hypospadias	MMF/MPA, cyclosporine
2015	2011	26	Hypoplastic toes	MMF/MPA, tacrolimus, prednisone
2015	2013	32	Urinary tract malformation	MMF/MPA, tacrolimus
2016	2010	32	Plagiocephaly	MMF/MPA, tacrolimus, prednisone
2017	2006	38	Wolff Parkinson White syndrome	MMF/MPA, tacrolimus, prednisone

*MMF/MPA* Mycophelonate mofetil/Mycophenolic acid, *AZA* Azathioprine

^a^ Age at conception of the pregnancy, in years

### Willingness of having a child in male transplanted patients under immunosuppressive therapy

In the cohort, 175 (13%, 95% CI [11–15]) participants reported having a desire to father a child in the future, while 140 (11%, 95% CI [9–12]) felt anxious about fathering a child while taking immunosuppressive medications. Of these 175 subjects, 50 (4%, 95% CI [3–5]) patients have had treatment modifications linked to their willingness to conceive. Table 6 describes the patients’ feelings regarding a future pregnancy according to their fatherhood status.

**Table 6:** Participants’ feelings regarding a future pregnancy according to their fatherhood status

Covariate	All patients (***n*** = 1332)	Patients without children after KT (***n*** = 1100)	Patients having fathered after KT (***n*** = 232)
Future desire to father a child, n (%, [95% CI])	175 (13%, [11–15])	119 (11%, [9–13])	56 (24%, [19–30])
Anxiety regarding a future pregnancy, n (%, [95% CI])	140 (11%, [9–12])	106 (10%, [8–12])	34 (15%, [10–20])
Treatment modification to prepare for pregnancy, n (%, [95% CI])	50 (4%, [3–5])	34 (3%, [2–4])	16 (7%, [4–11])

*KT* kidney transplant

## Discussion

Successful transplantation improves reproductive function, permitting male KT recipients to father children [22, 39, 40]. The impact of immunosuppressive medication on the fertility of male KT recipients and their offspring is however unknown, since data related to paternal exposure at the time of conception are sparse. We hereby provide an estimation, based on a survey, of the proportion of male KT recipients having fathered children, and the outcome of the pregnancies.

We report a paternity rate in our population of 17%, a majority of the participants did not have children after renal transplantation.

### Infertility issues

The reported infertility rate in our childless population has been estimated at 17% mainly because of spermatogenesis disorders, higher than that in the general population (Table 1). In France, among 10–15% of couples who suffer from infertility, approximately 20–30% have a male origin [41]. It is generally assumed that approximately 6–7% of men face fertility problems [42–44]. Immunosuppressive agents, notably sirolimus, have been associated with impaired fertility after transplant [25, 27–29]. In a study from Zuber et al. [29], the proportion of patients who had fathered after KT was significantly lower in patients treated with sirolimus, with abnormalities in sperm analysis. Our results highlight that 27% of patients who had fathered a child after KT needed medically assisted reproduction, with an equal proportion in each group of immunosuppressive agents. The issue of male fertility in kidney transplant recipients requires additional studies.

### Outcome of pregnancies fathered by male kidney transplant recipients under immunosuppressive therapy at the time of conception

This is to date one of the largest cohorts, apart from the NTPR reports, regarding paternity in transplanted patients [4, 5, 22]. In our population, 86, 9, 3 and 0.5% of respectively livebirths, spontaneous abortions, therapeutic abortions and stillbirths, occurred. The higher occurrence of unwanted outcomes observed in pregnancies fathered by patients treated with everolimus, sirolimus and belatacept should be interpreted with caution as only a few patients were exposed to these agents. Of note, the occurrence of spontaneous abortion in the general population varies between 12 and 15% of pregnancies [45]. In France, authorizations for therapeutic abortion are delivered in approximately 0.93% of pregnancies [46], a lower rate than the one observed in our population. The general population stillbirth rate varies from 0.9 to 0.4% [47], which is similar to our results.

In our study, among the 349 livebirths, only 9% were pre-term delivered, 6% had low birth-weights < 2.5 Kg, and 4% had malformations. It should be noted that there was a higher rate of pre-term delivery in pregnancies fathered by patients treated with everolimus, and a higher rate of low birth-weight in pregnancies fathered by patients treated with sirolimus. These results should be interpreted very cautiously as only 5 and 7 patients were treated with everolimus and sirolimus, respectively. In the general population, the pre-term delivery and low birth-weight rates are 7.5%, and birth defects occur in approximately 2–3% of newborns [48, 49].

Our study shows that there was no difference in the proportion of congenital malformations whether the father was exposed or not to immunosuppressive agents. Reports and analyses from the NTPR concluded that the outcomes of pregnancies fathered by male transplant recipients were similar to those of the general population. In 2010, Coscia et al. [5] reported 902 pregnancies fathered by 591 male KT recipients, with 93% livebirths but birth defects were not described. A Norwegian study reported 474 children born after transplantation, and no increased risk was found for any pregnancy outcomes compared with the general population, with a malformation rate of 1.9% [23]. Of the 13 congenital malformations in our population, 3 (one congenital phimosis, one pyloric stenosis and one Wolf Parkinson White syndrome) are well known malformations that have never been associated with any medication [50–52].

### Paternal exposure to MMF/MPA at the time of conception

There has been increasing concern since the EMA recommendations on the precautions male KT recipients should take while being treated with MMF/MPA [32, 33, 35–37]. These recommendations rely on the theoretical potential of chromosomal damage due to the transfer of MMF/MPA through seminal fluid [30, 35]. In our study, pregnancies were mostly conceived under the following medications: CTC, MMF/MPA and tacrolimus, which is currently the most common post-transplant combination worldwide [24]. The malformation rates were 4, 3 and 5% respectively, without highlighting any over-representation of malformations under MMF. The highest malformation rate (10%) was observed with azathioprine exposure. Jones et al. [22] identified 205 pregnancies fathered by male KT recipients exposed to MMF/MPA at the time of conception, and among 194 livebirths, the rate of malformations and prematurity were 3.1 and 11%, respectively. No specific pattern of malformation was identified [22]. Recently, Midtvedt et al. [24] retrospectively compared outcomes in pregnancies fathered by male KT recipients either exposed to MMF/MPA or not at the time of conception. There were 112 patients who fathered 155 children under MMF/MPA, and 133 patients who fathered 195 children without MMF/MPA exposure. They found no difference in malformations nor pre-term deliveries in children fathered by MMF/MPA exposed compared to KT recipients not exposed [24]. The outcomes of pregnancies fathered by male transplant recipients appear similar to those of the general population and MPA embryopathies have not been noted in pregnancies fathered by those patients [21, 53]. Our results add to the available data in the literature, that pregnancies fathered by patients under MMF/MPA at the time of conception do not seem to have more complications than pregnancies in the general population.

### Anxiety issues

To our knowledge, patient anxiety about the impact of immunosuppressive drugs on pregnancies and children has never been previously studied. Our survey reveals a signal of increased anxiety in this population, which is important to take into account to improve patient’s quality of life. Among participants who never had children, one-third did not have a desire to father children, without any differences across the centres. It remained a common feeling among men who wanted children after transplantation, as the childless patients reported feeling anxious about exposure to immunosuppressive drugs (Table 6). The design of the survey did not inquire about the reasons of this anxiety, nor its care. Our findings will require further qualitative studies, to better describe the sources of anxiety. A multidisciplinary transplant team, to help KT patients medically as well as psychologically, seems necessary in the light of our findings.

### Limitation of the survey

This study has several limitations, most notably the limited response rate which was less than 50% despite several relaunches to improve participation. Our population may not be representative of the experiences of all male KT recipients. Participation was voluntary and was thus subject to reporting and selection biases. Although the response rate may limit the generalizability of our results, this survey represents to our knowledge one of the largest sampling of pregnancies fathered by male KT recipients. It should also be noted that surveys may accentuate the concerns of a minority of responders, further limiting generalizability.

The use of a questionnaire to collect past events could introduce memory bias. Participants needed to recall events that could have happened over 10–15 years back in time, which could question the validity of the collected data.

Additionally, in addition to immunosuppressive agents, KT recipients are treated with multiple other medications, and thus exposures to various other agents cannot be excluded. As previously discussed, our spontaneous abortion rate is probably under-estimated due to a misunderstanding of the questionnaire by some patients. Information about immunosuppressive drugs of patients who did not father children after KT was not collected, which limits the interpretation of our infertility rate. Moreover, our study lacks information on the graft function, which also influences male KT recipients’ ability to conceive. Finally, because of the design of the study, medical information about the mother as well as her potential treatments were not available, even though the mothers’ exposure can have a major impact when studying pregnancy outcomes.

## Conclusion

In conclusion, our survey adds further information on the proportion of malformations in the offspring of transplanted men exposed to immunosuppressive drugs, which remains a crucial issue that has lacked evidence to date. Despite the limited response rate, this survey does not provide any warning signal that pregnancies fathered by male patients exposed to immunosuppressive agents, notably the debated MMF/MPA, have more complications than pregnancies in the general population; which is consistent with previous studies.

Even more data will be needed in the future; and health care providers are encouraged to report the outcomes of such pregnancies in the literature.

## Supplementary Information

**Additional file 1: Supplementary data. Figure S1.** Questionnaire. **Table S1.** Number of included patients and participants according to the centre. **Table S2.** Male kidney transplant recipients’ characteristics according to their paternity status.

## Data Availability

All data generated or analysed during this study are available from the corresponding author on reasonable request.

## Abbreviations

95% CI: 95% confidence interval
ANSM: Agence Nationale de la Santé et des Médicaments
AZA: Azathioprine
CTC: Corticosteroid
EMA: European Medicines Agency
IQR: Interquartile range
Kg: Kilogram
KT: Kidney transplant
MMF/MPA: Mycophenolate mofetil/mycophenolic acid
NA: Non-applicable
NTPR: National Transplantation Pregnancy Registry
PKD: Polycystic kidney disease
