# Supplementary material for: Paternity in male kidney transplant recipients: a French national survey, the PATeRNAL study
Source: BMC Nephrol. 2020 Nov 16;21:483. doi: 10.1186/s12882-020-02115-x (PMC7667842; doi:10.1186/s12882-020-02115-x)
Supplement: Supplementary file 1 — Additional file 1: Supplementary data. Figure S1. Questionnaire. Table S1. Number of included patients and participants according to the centre. Table S2. Male kidney transplant recipients’ characteristics according to their paternity status. [file 12882_2020_2115_MOESM1_ESM.docx]

**Supplementary data**

| Year (s) of kidney transplant (s) |  | (1^e^) …… (2^e^) …………….. |  | (3^e^) ………… (4^e^) ……………. |
| --- | --- | --- | --- | --- |
| Your current age (years): ………. |  |  |  |  |
| Do you have children: Yes No | | | | |
| Number of children:  Before kidney transplant: ……………………. After kidney transplant: ……………………. | | | | |
| If you didn’t have children after kidney transplant, what was the reason?  - You did not wish to have a child: No Yes  - Inability to have a child: No Yes. If yes, please specify: ………………………………………………………………………………………  - Anxiety about fathering a child under immunosuppressive medication: Yes No  - Other: ………………………………………………………………………………………………………………………………………………………………………………………………. | | | | |

**Figure S1.** Questionnaire

| **Pregnancy (ies) of your partner after kidney transplantation** | **1^st^ pregnancy** | |  | | **2^d^ pregnancy** | |  | | **3^d^ pregnancy** | |  |
| --- | --- | --- | --- | --- | --- | --- | --- | --- | --- | --- | --- |
| Year of pregnancy (YYYY) | …. | |  | | …. | |  | | …. | |  |
| Did you and/or your partner consult reproductive health services? | Yes No | |  | | Yes No | |  | | Yes No | |  |
| Were you taking Cellcept^®^ or Myfortic^®^? | Yes No | |  | | Yes No | |  | | Yes No | |  |
| Were you taking Imurel^®^? | Yes No | |  | | Yes No | |  | | Yes No | |  |
| Were you taking Tacrolimus (Prograf^®^, or Advagraf^®^, or Adoport^®^, or Envarsus^®^)? | Yes No | |  | | Yes No | |  | | Yes No | |  |
| Were you taking ciclosporine (Neoral^®^, Sandimmun^®^)? | Yes No | |  | | Yes No | |  | | Yes No | |  |
| Were you taking Rapamune^®^? | Yes No | |  | | Yes No | |  | | Yes No | |  |
| Were you taking Certican^®^? | Yes No | |  | | Yes No | |  | | Yes No | |  |
| Were you taking Nulojix^®^? | Yes No | |  | | Yes No | |  | | Yes No | |  |
| Were you taking steroids (Cortancyl^®^, Prednisone^®^)? | Yes No | |  | | Yes No | |  | | Yes No | |  |
| Did you suffer from graft rejection before your partner’s pregnancy? | Yes No  I don’t know | |  | | Yes No  I don’t know | |  | | Yes No  I don’t know | |  |
| Did you receive any of these treatments before your partner’s pregnancy: Rituximab^®^, Endoxan^®^, plasma exchanges, immunoglobulins? | Yes No  I don’t know | |  | | Yes No  I don’t know | |  | | Yes No  I don’t know | |  |
| Did your partner have a miscarriage? | Yes No | |  | | Yes No | |  | | Yes No | |  |
| Did your partner have a therapeutic abortion? | Yes No | |  | | Yes No | |  | | Yes No | |  |
| **After pregnancy** | | **1^st^ child** | |  | | **2^d^ child** | |  | | **3^d^ child** | |
| Date of birth (MM/YYYY) | | ../…. | |  | | ../…. | |  | | ../…. | |
| Length of pregnancy: weeks (W) or months (M) | | ……………… | |  | | ……………… | |  | | ……………… | |
| Weight at birth (Kg) | | ……………… | |  | | ……………… | |  | | ……………… | |
| Baby’s sex | | Male Female | |  | | Male Female | |  | | Male Female | |
| The baby was healthy? | | Yes No | |  | | Yes No | |  | | Yes No | |
| The baby was stillborn? | | Yes No | |  | | Yes No | |  | | Yes No | |
| The baby had malformations? | | Yes No  If yes, please specify: ……………………… | |  | | Yes No  If yes, please specify: ……………………… | |  | | Yes No  If yes, please specify: ……………………… | |
| Does your child suffer from intellectual disability? | | Yes No | |  | | Yes No | |  | | Yes No | |

**Plans for the future :**

-Do you wish to have a child in the future?

Yes No

-Are you anxious about fathering a child under immunosuppressive medication?

Yes No

-If so, have you considered with your nephrologist to adapt your immunosuppressive medication in order to father a child?

Yes No

**Table S1.** Number of included patients and participants according to the centre

| Center | Included patients n=2908 | Participants n=1332 (46%) |
| --- | --- | --- |
| Centre 1 | 186 | 87 (47%) |
| Centre 2 | 171 | 91 (53%) |
| Centre 3 | 178 | 76 (43%) |
| Centre 4 | 206 | 101 (49%) |
| Centre 5 | 168 | 85 (51%) |
| Centre 6 | 127 | 59 (47%) |
| Centre 7 | 500 | 214 (43%) |
| Centre 8 | 191 | 92 (48%) |
| Centre 9 | 137 | 42 (31%) |
| Centre 10 | 222 | 112 (51%) |
| Centre 11 | 217 | 114 (53%) |
| Centre 12 | 282 | 121 (43%) |
| Centre 13 | 323 | 138 (43%) |

Results are expressed as number (percentage)

- **Table S2.** Male kidney transplant recipients’ characteristics according to their paternity status

| **Covariate** | **All patients (n=1332)** | **Childless patients (n=330)** | **Patients having fathered before KT only* (n=770)** | **Patients having fathered after KT  (n=232)** |
| --- | --- | --- | --- | --- |
| Age, median (IQR), years | 55 (46-62) | 50 (39-59) | 59 (53-64) | 42 (36.3-48) |
| Year of first transplantation, median (IQR) | 2008 (2006-2012) | 2009 (2005-2012) | 2009 (2007-2012) | 2006 (1998-2010) |
| Year of second transplantation, median (IQR) | 2009 (2006-2013) | 2008 (2005-2011) | 2010 (2007-2014) | 2009 (2007-2013) |
| Centre |  |  |  |  |
| Centre 1 | 87 (7%) | 19 (6%) | 51 (7%) | 17 (7%) |
| Centre 2 | 91 (7%) | 16 (5%) | 61 (8%) | 14 (6%) |
| Centre 3 | 76 (6%) | 28 (9%) | 38 (5%) | 10 (4%) |
| Centre 4 | 101 (8%) | 22 (7%) | 66 (8%) | 13 (6%) |
| Centre 5 | 85 (6%) | 27 (8%) | 48 (6%) | 10 (4%) |
| Centre 6 | 59 (4%) | 14 (4%) | 32 (4%) | 13 (6%) |
| Centre 7 | 214 (16%) | 52 (16%) | 118 (15%) | 44 (19%) |
| Centre 8 | 92 (7%) | 27 (8%) | 55 (7%) | 10 (4%) |
| Centre 9 | 42 (3%) | 8 (2%) | 21 (3%) | 13 (6%) |
| Centre 10 | 112 (8%) | 23 (7%) | 63 (8%) | 26 (11%) |
| Centre 11 | 114 (9%) | 32 (10%) | 65 (8%) | 17 (7%) |
| Centre 12 | 121 (9%) | 31 (9%) | 68 (9%) | 22 (10%) |
| Centre 13 | 138 (10%) | 31 (9%) | 84 (11%) | 23 (10%) |
| Number of children, median (IQR) | 2 (1-2) | NA | 2 (2-3) | 2 (1-2) |
| Child before transplantation, n (%) | 842 (63%) | NA | 770 (100%) | 72 (31%) |
| Child after transplantation, n (%) | 232 (17%) | NA | 0 (0%) | 232 (100%) |

IQR: Interquartile range; NA: non applicable
* Male kidney transplant recipients having fathered children before transplantation only, no children after transplantation
